# Coupling dairy wastewaters for nutritional balancing and water recycling: sustainable heterologous 2-phenylethanol production by engineered cyanobacteria

**DOI:** 10.3389/fbioe.2024.1359032

**Published:** 2024-03-01

**Authors:** Giulia Usai, Alessandro Cordara, Elena Mazzocchi, Angela Re, Debora Fino, Candido Fabrizio Pirri, Barbara Menin

**Affiliations:** ^1^ Centre for Sustainable Future Technologies, Fondazione Istituto Italiano di Tecnologia, Turin, Italy; ^2^ Department of Applied Science and Technology—DISAT, Politecnico di Torino, Turin, Italy; ^3^ Department of Environment, Land and Infrastructure Engineering—DIATI, Politecnico di Torino, Turin, Italy; ^4^ Istituto di Biologia e Biotecnologia Agraria, Consiglio Nazionale delle Ricerche IBBA-CNR, Milan, Italy

**Keywords:** cyanobacteria, heterologous production, photosynthesis, 2-phenylethanol, dairy wastewater, water recycling, phycoremediation

## Abstract

Microalgae biotechnology is hampered by the high production costs and the massive usage of water during large-volume cultivations. These drawbacks can be softened by the production of high-value compounds and by adopting metabolic engineering strategies to improve their performances and productivity. Today, the most sustainable approach is the exploitation of industrial wastewaters for microalgae cultivation, which couples valuable biomass production with water resource recovery. Among the food processing sectors, the dairy industry generates the largest volume of wastewaters through the manufacturing process. These effluents are typically rich in dissolved organic matter and nutrients, which make it a challenging and expensive waste stream for companies to manage. Nevertheless, these rich wastewaters represent an appealing resource for microalgal biotechnology. In this study, we propose a sustainable approach for high-value compound production from dairy wastewaters through cyanobacteria. This strategy is based on a metabolically engineered strain of the model cyanobacterium *Synechococcus elongatus* PCC 7942 (already published elsewhere) for 2-phenylethanol (2-PE). 2-PE is a high-value aromatic compound that is widely employed as a fragrance in the food and cosmetics industry thanks to its pleasant floral scent. First, we qualitatively assessed the impact of four dairy effluents on cyanobacterial growth to identify the most promising substrates. Both tank-washing water and the liquid effluent of exhausted sludge resulted as suitable nutrient sources. Thus, we created an ideal buffer system by combining the two wastewaters while simultaneously providing balanced nutrition and completely avoiding the need for fresh water. The combination of 75% liquid effluent of exhausted sludge and 25% tank-washing water with a fine-tuning ammonium supplementation yielded 180 mg L^−1^ of 2-PE and a biomass concentration of 0.6 gDW L^-1^ within 10 days. The mixture of 90% exhausted sludge and 10% washing water produced the highest yield of 2-PE (205 mg L^−1^) and biomass accumulation (0.7 gDW L^−1^), although in 16 days. Through these treatments, the phosphates were completely consumed, and nitrogen was removed in a range of 74%–77%. Overall, our approach significantly valorized water recycling and the exploitation of valuable wastewaters to circularly produce marketable compounds via microalgae biotechnology, laying a promising groundwork for subsequent implementation and scale-up.

## 1 Introduction

Mass microalgae cultivation requires colossal volumes of water. For instance, microalgae have the largest water footprint among biofuel feedstocks (Zhang et al., 2014). As a possible strategy, reusing the cultural medium for microalgae production strongly reduces freshwater demand and nutrient usage, limiting the concerns about these cultivations (Yang et al., 2011). However, reused water may lead to accumulated cell exudates, salts, bacteria, viruses, and cell debris, which can potentially inhibit microalgae (Farooq et al., 2015). Notably, water is also an essential resource in almost all industrial sectors, including pharmaceutics, electronics, food and beverage, textile, petrochemicals, agrochemicals, and oil and gas, and domestic purposes (Wollmann et al., 2019; Abdelfattah et al., 2023). The resulting effluents contain different contaminants, such as organic carbon (Venkata Mohan et al., 2008), nitrogen (Carrera et al., 2003), phosphorous (Bunce et al., 2018), salts (Zhao et al., 2020), heavy metals (Barakat, 2011), and solid organic matter, and must be treated prior to disposal. Therefore, new technologies aimed at removing pollutants and valorizing wastewaters are needed. For instance, the dairy industry utilizes large water volumes for its activities (e.g., cleaning equipment, milk processes, pasteurization, and sterilization), and it has been estimated that 1–10 m^3^ of water per m^3^ of processed milk is employed in this sector (Boguniewicz-Zablocka et al., 2019). In 2019, Europe was the second largest producer of milk, with 167.4 million tons (FAO, 2019) (FAO, 2020). The production of wastewater calculated for liquid milk, cheeses, butterfat, and fermented milk products (83% of dairy products) was 192.5 × 10^6^ m^3^ per year (Stasinakis et al., 2022). Particularly, Italy is the third European country counting 18.9 × 10^6^ m^3^ of dairy effluents in 2019 (Stasinakis et al., 2022). The most abundant components are fats, organic compounds, proteins, and minerals, with chemical oxygen demand (COD) ranging from 80 to 95 g L^−1^ and a biochemical oxygen demand (BOD) of 40–48 g L^−1^ (Qasim and Mane, 2013). Additionally, dairy wastewaters contain large amounts of total nitrogen and total phosphorus (14–830 mg L^−1^ and 9–280 mg L^−1^, respectively), while the pH ranges from very acidic to alkaline (Chokshi et al., 2016). Thus, different kinds of liquid wastes are produced. Among these, the most biologically investigated by-product for its recovery and valorization is cheese whey (CW) (Atra et al., 2005; Zandona et al., 2021; Sar et al., 2022). CW is a nutrient-rich by-product and is derived from milk coagulation and the subsequent separation of curd; it is estimated that about 8–9 kg of CW is produced for every 1–2 kg of cheese product (Baldasso et al., 2011). Moreover, due to the content of phosphorus (0.124–0.54 kg m^−3^) and nitrogen (0.2–1.76 kg m^−3^) (Prazeres et al., 2012), CW has a high eutrophication risk for the receiving environments (Carvalho et al., 2013), but it is biotechnologically exploitable (Carvalho et al., 2013). However, in Italy, CW itself represents a resource for the cheese industry since it is employed to produce ‘Ricotta’ cheese, whose relative waste is called ‘scotta’ (Sc) (Maragkoudakis et al., 2016). Scotta is characterized by high COD and BOD (Sansonetti et al., 2009; Bergamaschi et al., 2016; Monti et al., 2018), and it is environmentally dangerous if not properly processed (Ribeiro et al., 2017). In addition, the large volume of effluents generated during the various production steps related to the cleaning of tanks and pipelines should be considered. The washing procedures account for 75% of the total water used by the dairy industry (Buabeng-Baidoo et al., 2017). These washing waters (WWs) retain significant quantities of dissolved nutrients, proteins, fats, and lactose (Brião et al., 2019). Finally, the secondary wastewaters of the dairy industry are derived from the biological treatments of raw or primary dairy effluents, e.g., activated sludge treatment. Currently, it is applied as the main strategy for the removal of organic compounds and nitrogen from dairy effluents (Lateef et al., 2013). The resulting exhausted sludge (ES) still contains contaminants, mainly nitrogen and phosphorus, as well as metals (Kaur, 2021), and requires further processing as tertiary treatments (Zagklis and Bampos, 2022). In this scenario, microalgae and cyanobacteria bioremediation, also known as phycoremediation, suits perfectly. Phycoremediation is a consolidated approach that not only exploits challenging wastewaters but also benefits the bioeconomy by providing value-added products, i.e., pigments, lipids, carbohydrates, proteins, and bioactive pharmaceutical metabolites (Kalra et al., 2021; Uma et al., 2023). The advantage of choosing microalgae or cyanobacteria for this purpose lies in their capability to grow on a variety of industrial effluents. These microorganisms photosynthesize by absorbing CO_2_ from the atmosphere and use some of the pollutants found in wastewaters as nutrients (Ansari et al., 2017). Particularly, a plethora of microalgal species, such as *Chlorella pyrenoidosa*, *Anabaena ambigua*, *Scenedesmus abundans*, *Chlorella vulgaris*, *Chlorella sorokiniana*, *Chlamydomonas polypyrenoideum*, and *Acutodesmus dimorphus*, have been successfully cultivated in wastewaters from the dairy industry (Kothari et al., 2013; Qin et al., 2014; Choi, 2016; Chokshi et al., 2016; Brar et al., 2019; Kusmayadi et al., 2022). In all these studies, COD and BOD were reduced up to 90%, and nitrogen and phosphates were reduced by 70%–90% and 80%–100%, respectively. Eukaryotic microalgae biomass produced from these treatments is usually exploited as feedstock for biofuel production since the biomass can contain up to 40%–70% lipids (Kothari et al., 2013; Chokshi et al., 2016; Khalaji et al., 2023; Paulenco et al., 2023). On the other hand, cyanobacteria, which constitute a group of ancient ubiquitous phototrophic bacteria (Abed et al., 2009; Garcia-Pichel, 2009), are resistant to extreme conditions of temperature, pH, heavy metals, and high salinity and, thus, are suitable for bioremediation (Gaurav et al., 2018; Ahmad, 2022; Lakmali et al., 2022). Although cyanobacteria have the ability to successfully remove nitrogen and phosphate from wastewaters (Lincoln et al., 1996; Kumar et al., 2011; El-Sheekh et al., 2011; Jitha and Madhu, 2016; Kabariya and Ramani, 2016; Ouhsassi et al., 2020; Álvarez et al., 2020; Ahmad, 2022), they have been mainly investigated for the production of high-value compounds, both naturally produced and metabolically engineered, thanks to their ease of genetic manipulation. These compounds can be extruded or derivatives of biomass, such as pigments (Kabariya and Ramani, 2018; Menin et al., 2019; Arashiro et al., 2020; Thevarajah et al., 2023). Alternatively, cyanobacteria have been exploited for disinfection treatment of waste effluents (Samiotis et al., 2023), and their biomass has been used as a substrate for fermentation processes (Möllers et al., 2014; Comer et al., 2020; Álvarez et al., 2020; De Oliveira et al., 2022). Thus, exploiting nutrient-rich wastewaters as growth substrates not only facilitates the cultivation of microalgae and cyanobacteria but also serves the dual purpose of contaminant removal and reduction in freshwater usage. Consequently, this approach plays a pivotal role in cost-cutting within the realm of microalgae and cyanobacteria biotechnology.

Here, we aimed to develop a sustainable heterologous production of the high-value compound 2-phenylethanol (2-PE) by a metabolically engineered strain of *Synechococcus elongatus* PCC 7942, known as 2PE_*aroK* (Usai et al., 2022). 2-PE is an aromatic alcohol with a pleasant rose-like scent, which finds application mostly in the food, fragrance, and flavor industries (Etschmann et al., 2002; Nielson et al., 2018; Fang and Sung, 2021). The 2PE_*aroK* recombinant strain is able to overexpress five genes: *aroG* ^*fbr*^ *and pheA* ^*fbr*^ (from *E. coli*), *aroK* (native), related to the shikimate pathway, the metabolic route for aromatics production (Gosset, 2009); *kivD* (*L. lactis*) and *adhA* (*Synechocystis* PCC 6803), responsible for the final synthesis steps (Supplementary Table S1). The 2PE_*aroK* has been extensively described by Usai et al. (2022). The herein proposed sustainable 2-PE bioproduction is tailor-made by coupling wastewaters from the dairy industry, which allowed to balance the pH and nutritional contributions, avoiding the use of freshwater and resulting in the successful production of 2-PE.

## 2 Materials and methods

### 2.1 Pre-treatment of dairy wastewaters

All the dairy waste streams were first subjected to natural sedimentation (overnight at 4°C) in order to eliminate the solid particulate. The liquid effluent of the exhausted sludge was filtered with polyethersulfone filtration systems (pore size 0.22 µm). The effluents were stored at 4°C until use. Cheese whey, scotta, and the tank-washing water were deproteinized (105°C; 5 min), and the protein aggregates were left to sediment and stored at 4°C until use. To prevent biological contamination, almost all the preparative steps were conducted at 4°C, rigorously avoiding frequent thermal changes of the effluents.

### 2.2 Hydrochemical analysis of dairy wastewaters

The pre-treated dairy wastewaters were shipped to E.L.A. s.r.l. (Asti, Italy) for hydrochemical analysis. The following parameters were determined: pH and conductivity, COD, BOD, total nitrogen, and total phosphorous. Additionally, NH₄⁺, NO_3_^−^, PO₄³⁻, SO_4_^2−^, and dissolved metal ions (Ca, Na, Cl, K, Fe, Al, As, Ba, Hg, Se, Mn, Ni, Cr, Cd, and Cu) were determined. The methods utilized are reported in Table 1.

**TABLE 1:** Chemical analysis of four dairy wastewaters: cheese whey, scotta, washing waters, the liquid effluent of the exhausted sludge. The data are expressed as mg L^−1^. The analytical methods used for their quantification are reported in the last column.

Description	Cheese whey	Scotta	Washing waters	Exhausted sludge	Analytical method
***COD (mgO***_***2***_ ***L*** ^***-1***^ ***)***	48,600	53,800	5,308	22	ISO 15705:2002
***BOD (mgO***_***2***_ ***L*** ^***-1***^ ***)***	20,100	22,300	2,200	< 10	M.I. 21 Rev. 0:2013
***Total nitrogen***	475	466	166.8	5.91	
***Ammonical nitrogen***	103	3.9	< 0.05	83	UNI 11669:2017
***Nitric nitrogen***	< 1.5	< 1.5	< 1.5	< 1.5	APAT CNR IRSA 4020 Man 29 2003
***Phosphates***	649	1,090	69.5	100	
***Sulfates***	113	117	6.69	< 5	
***Chloride***	7,090	9,610	54.4	287	
***Total phosphorous***	249	382	27.8	33.4	UNI EN ISO 15587–1:2002 (Annex A) + UNI EN ISO 11885:2009
***Na***	3,000	4,470	29.8	414	
***Ca***	161.048	418.47	44.311	48.727	
***K***	1,120	1,220	44.2	61	
***Al***	0.047	0.062	0.03	0.021	
***As***	0.03	0.029	< 0.01	< 0.01	
***Fe***	0.059	0.077	0.042	0.06	
***Mn***	< 0.01	< 0.01	< 0.01	0.02	
***Cu***	0.011	0.018	0.015	< 0.01	
***Se***	0.016	0.029	0.012	< 0.01	
***Ba***	0.011	< 0.01	< 0.01	< 0.01	
***Cd***	< 0.01	< 0.01	< 0.01	< 0.01	
***Cr***	< 0.01	< 0.01	< 0.01	< 0.01	
***Hg***	< 0.01	< 0.01	< 0.01	< 0.01	
***Ni***	< 0.01	< 0.01	< 0.01	< 0.01	
***Pb***	< 0.01	< 0.01	< 0.01	< 0.01	
***pH***	5.78	3.84	4.57	8.43	APAT CNR IRSA 2060 Man 29 2003
***Conductivity (mS/cm)***	18.83	25.87	0.53	3.02	UNI EN 27888:1995

### 2.3 Cyanobacterial strain and culture maintenance

All the tests reported in the present investigation were conducted using a metabolically engineered strain of *S. elongatus* PCC 7942, known as 2PE_*aroK*, already developed by our laboratory (Usai et al., 2022). This strain is able to heterologously produce 2-PE, and Supplementary Table S1 reports all the genes and relative proteins involved in this pathway; they were also thoroughly described by Usai et al. (2022). The mutant strain was grown in BG11 (Cordara et al., 2018; Vasile et al., 2021) as a standard medium to define the control condition of the qualitative screening. In brief, the present BG11 medium is composed as follows: 1.5 g L^−1^ NaNO_3_, 0.075 g L^−1^ MgSO_4_·7H_2_O, 0.004 g L^−1^ FeCl_3_·6H_2_O, 0.04 g L^−1^ K_2_HPO_4_, 0.036 g L^−1^ CaCl_2_, 0.024 g L^−^1 Na_2_EDTA·2H_2_O, 2.86 mg L^−1^ H_3_BO_3_, 1.81 mg L^−1^ MnCl_2_·4H_2_O, 0.39 mg L^−1^ Na_2_MoO_4_·2H_2_O, 0.22 mg L^−1^ ZnSO_4_·7H_2_O, 0.05 mg L^−1^ CuSO_4_·5H_2_O, and 0.03 mg L^−1^ Co(NO_3_)_2_·6H_2_O. The BG11 medium was buffered with 6.05 g L^−1^ TES (2-([Tris(hydroxymethyl)methyl]amino)ethane-1-sulfonic acid sodium salt) to pH = 8. The BG11 medium was supplemented with 20 μg mL^−1^ spectinomycin and 10 μg mL^−1^ kanamycin. All the cyanobacteria cultures were grown at 30°C, 130 rpm, and continuous illumination of 30 μmol photons m^−2^ s^−1^.

### 2.4 Cyanobacteria growth conditions on dairy wastewaters

All the experiments were performed at the same environmental parameters: 30°C, 130 rpm, a continuous light intensity of 30 μmol photons m^−2^ s^−1^, and with 20 μg mL^−1^ spectinomycin and 10 μg mL^−1^ kanamycin.

**FIGURE 1:**
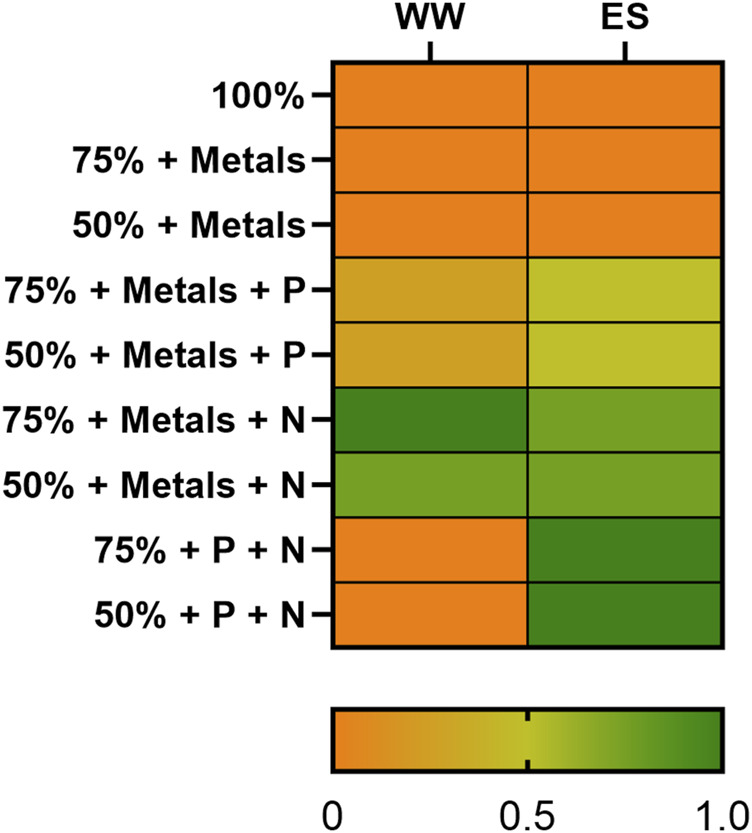
Dairy wastewater qualitative screening. The screening was conducted as biological triplicates for each tested condition against a control one (BG11). The study considered the effect on the growth of the 2PE_*aroK S. elongatus* PCC 7942 mutant strain, ranging from no growth (orange) to a growth equal to the control condition (dark green). The percentages indicate the ratio of each waste used in the single experiment.

To evaluate the qualitative effect of the dairy wastewaters on the mutant strain of cyanobacteria, the bacterial growth was evaluated on 100, 75, and 50% of singular wastewater using 6-well plates. Moreover, different supplementations were assessed in order to better understand the limiting nutritional factor associated with each waste and highlight the most potential growth conditions in dairy wastewaters (Figure 1). For this purpose, trace element solution, nitrate, or phosphates were supplemented singularly or in combination at the same concentration reported for the BG11 medium. Then, the combination of ES and WW was studied at different ratios: 90:10, 75:25, and 60:40. The pH tolerance of the 2PE_*aroK* mutant strain on the previously selected conditions was evaluated. To assess the pH buffering system in ES and WW, the MOPS (4-(3-sulfonatopropyl)morpholin-4-ium) buffer was adopted (25, 50, and 75 mM) using TES as the control buffer (Supplementary Table S2). The final physiological characterization of the mutant strain on 90:10 and 75:25 mixtures of ES and WW was performed in 250-mL Erlenmeyer flasks in the abovementioned parameters. All the experiments were conducted using a starting inoculum equivalent to OD_730_ = 0.2. The growth of the 2PE_*aroK* mutant strains of *S. elongatus* PCC 7942 was monitored by measuring the optical density at 730 nm (OD_730_) and then converted into biomass as a gram of dry cell weight per liter (gDW L^-1^) using a conversion factor of 0.26 [previously developed in our laboratory (Usai et al., 2022)]. The specific growth rate (μ, d^−1^) was obtained from the slope of the logarithmic plot of OD_730_ versus time for the growth curve points belonging to the exponential phase (Table 2). The gene expression was induced by adding 1mM isopropyl β-d-1-thiogalactopyranoside (IPTG) into the proper growth condition, and the metabolite doping was launched with 0.3 g L^-1^ L-phenylalanine (L-Phe).

**TABLE 2:** Summary of the main results of the present investigation about the growth of the 2PE_*aroK* mutant strain in 75:25 and 90:10 ratios of ES and WW. SD, standard deviation. The statistical tests were performed as the unpaired *t*-test (α ≤ 0.05) versus the control condition. *, *p*-value ≤0.05; **, *p*-value ≤0.002; ***, *p*-value ≤0.0002; and ****, *p*-value ≤0.0001. Chlα, chlorophyll α. Car, carotenoids.

		µ (d^-1^)			Max 2-PE titer (mg L^-1^)			Max 2-PE yield (mg gDW^-1^)			Average 2-PE productivity (mg L^-1^ d^-1^)			Chlα (mg gDW^-1^)			Car (mg gDW^-1^)		
Ratio ES:WW	Condition	Average	SD	*p*-value	Average	SD	*p*-value	Average	SD	*p*-value	Average	SD	*p*-value	Average	SD	*p*-value	Average	SD	*p*-value
**75:25**	***CTRL***	0.168	0.003		18.9	0.4		53.30	8.32		1.89	0.04		9.35	0.89		8.54	0.84	
	***I***	0.199	0.002	***	81.0	9.8	***	221.22	27.73	***	8.10	0.98	*	8.67	0.79	ns	6.42	0.33	*
	***II***	0.223	0.015	**	158.1	32.5	**	295.41	20.28	****	15.81	3.25	***	10.81	1.49	ns	5.16	0.25	*
	***III***	0.207	0.006	***	180.3	15.6	****	370.53	23.75	****	18.03	1.56	**	11.59	1.96	ns	5.45	0.34	*
**90:10**	***CTRL***	0.085	0.003		38.12	4.80		119.37	8.27		2.38	0.30		16.75	2.18		3.60	0.25	
	***I***	0.103	0.006	**	60.39	7.04	*	119.59	14.93	ns	3.77	0.44	*	18.70	1.43	ns	3.24	0.38	ns
	***II***	0.106	0.005	**	205.10	29.73	**	294.00	14.22	****	12.82	1.86	***	13.74	1.11	ns	2.32	0.04	**
	***III***	0.119	0.002	***	202.01	5.19	****	289.08	12.78	****	12.63	0.32	****	14.24	1.01	ns	2.34	0.16	**

### 2.5 Photosynthetic pigment quantification

Chlorophyll α (chlα) and total carotenoids (car) were quantified. An amount of 1 mL of cyanobacterial culture was harvested at the end of each test at approximately 75:25 and 90:10 ratios. The culture was pelleted 12,000 g for 10 min, and the supernatant was discharged. The cell pellet was resuspended in 1 mL of cold methanol (≥99.9%) and incubated at 4°C in the darkness for 20 min. The lysed cells were centrifuged again (12,000 g, 10 min, and 4°C) to recover the supernatant, which was properly diluted in methanol.

The spectrophotometric analyses were carried out on a PerkinElmer Lambda 650 UV/Vis spectrophotometer using quartz cuvettes. The instrument was calibrated using methanol as the blank. Each run was performed from 800 nm to 400 nm with a slit width of 1 nm.

To calculate the photosynthetic pigments concentration, the following equations were used (Wellburn, 1994):

$$\text{Chlorophyll }\alpha \;\left[\mu g\;{\text{mL}}^{-1}\right]=12.9447\left(A_{665}\;–\;A_{720}\right),$$

$$\text{Chlorophyll }\alpha \;\left[\mu M\right]=14.4892\left(A_{665}\;–\;A_{720}\right),$$

$$\text{Total carotenoids }\left[\mu g\;{\text{mL}}^{-1}\right]=\left[1000\left(A_{470}\;–\;A_{720}\right)\;–\;2.86\left(\text{Chla}\left[\mu g\;{\text{mL}}^{-1}\right]\right)\right]/221.$$

### 2.6 2-PE and L-Phe HPLC analysis

2-PE and L-Phe were quantified as previously reported (Usai et al., 2022). In brief, 2-PE production was assessed by ultrahigh-performance liquid chromatography (UHPLC UltiMate 3000, Thermo Fisher Scientific, Waltham, Massachusetts, United States) equipped with a Hypersil GOLD™ C18 Reversed Phase Column (250 × 4.6 mm, 5 μm; Thermo Fisher Scientific, Waltham, Massachusetts, United States); acetonitrile and H_2_O (50:50) were used with 1 mL min^-1^ flow rate at 40°C and UV-detection at 205 nm. L-phenylalanine was quantified through the Hypersil GOLD™ Amino column (120 × 4.6 mm, 5 μm; Thermo Fisher Scientific, Waltham, Massachusetts, United States) at 210 nm, and 9 mM sulfuric acid was pumped at 0.8 mL min^-1^; column oven was heated at 50°C. For sample preparation, 1 mL of bacterial culture was collected and centrifuged at 12,000 g for 10 min, and 20 μL of the supernatant was injected for HPLC analysis. 2-PE and L-Phe were both externally calibrated by using HPLC-grade standards (Sigma-Aldrich Inc., St. Louis, MO, United States).

## 3 Results

### 3.1 Selection of proper dairy wastewaters for *S. elongatus* growth

Here, the metabolically engineered strain of *S. elongatus* PCC 7942 2PE_*aroK* (Supplementary Table S1) was adopted to design a sustainable heterologous 2-phenylethanol production based on the exploitation of dairy wastewaters.

In this investigation, a chemical analysis and screening of the dairy effluents provided by a local Italian cheese factory were conducted first to evaluate the possibility of specifically cultivating the 2-PE-producing mutant strain. A detailed description is provided in the following sections.

#### 3.1.1 Chemical characterization of dairy wastewaters

Four dairy wastewater samples from the cheese production plant were analyzed: i) cheese whey (from cheese production), ii) scotta (from ricotta cheese production), iii) the washing water of pipelines and tanks, and iv) the liquid effluent of the exhausted sludge (coming from the biological treatment of the previous effluents). Table 1 shows the chemical characterization of the dairy streams. In the literature, for dairy effluents, the ratio of the key macroelements is reported to be C/N/P ≈ 200/3.5/1, which is generally considered nitrogen deficient to drive any biological processes (Prazeres et al., 2012).

#### 3.1.2 Nitrogen and phosphates

Nitrate (NO_3_^−^) and free ammonium (NH_4_^+^) are preferentially assimilated as nitrogen sources in almost all microorganisms (Wang et al., 2016) since nitrogen is an essential macronutrient needed for the biosynthesis of amino acids, proteins, and nucleic acids. The NO_3_^−^ concentration in the present wastewaters was <1.5 mg L^−1^ in all the waste streams, and the highest ammonium concentrations were 83 and 103 mg L^−1^ for ES and CW, respectively.

Here, the phosphate concentration, as well as the total phosphorous (TP), was high in all the wastewaters compared with the standard medium for cyanobacteria (which contains approx. 20 mg L^−1^ PO_4_^3-^) and higher than what should be in marine and freshwater bodies for identifying them as high quality and non-polluted. For these reasons, the present dairy wastewaters are great candidates for cyanobacteria treatments prior to disposal into the environment.

#### 3.1.3 Sodium, chloride, and calcium

Often, some cheese producers add NaCl or CaCl_2_ for specific cheese preparations (Guinee, 2004). Hence, high salinity is a peculiarity of the dairy streams, especially in the primary wastes. Here, CW and Sc had the highest salinity among the dairy streams (Table 1; conductivity 18.83 and 25.87 mS/cm, respectively).

#### 3.1.4 Metals

In prokaryotic and eukaryotic microalgae, copper (Cu) performs a pivotal role in either photosynthesis or respiratory electron transport (López-Maury et al., 2012). In the dairy wastewaters (Table 1), the Cu content appears stable in the range of 11–18 μg L^−1^, which is near to the content in the BG11 medium, except for ES, where Cu was not detected. Furthermore, iron (Fe) is fundamental for photosynthetic microorganisms since it is a cofactor for all three photosynthetic electron transfer chain complexes (Shcolnick and Keren, 2006). Even in this case, the Fe concentration in all the streams ranged 42–77 μg L^−1^, which is lower than that in the standard BG11 medium.

#### 3.1.5 pH, COD, and BOD

The range of pH associated with dairy wastewaters is wide. Here, the lowest pH was recorded for Sc (pH = 3.84), and the highest one was for ES (pH = 8.43). Generally, the optimum pH for cyanobacteria is 8, but it should never be as low as 7. Consequently, concerning the pH values, WW, CW, and Sc were not putatively suitable for cyanobacteria cultivation. Additionally, the high COD and BOD represent a huge challenge for the applicability of non-diluted CW and Sc caused by the rich content of organic carbon compounds. For high COD wastes, a preliminary anaerobic digestion or activated sludge treatment (as in this specific case for secondary treatment) is usually adopted prior to any other downstream application or disposal (Kusmayadi et al., 2022).

### 3.2 Qualitative screening of dairy wastewaters for cyanobacterial growth

**FIGURE 2:**
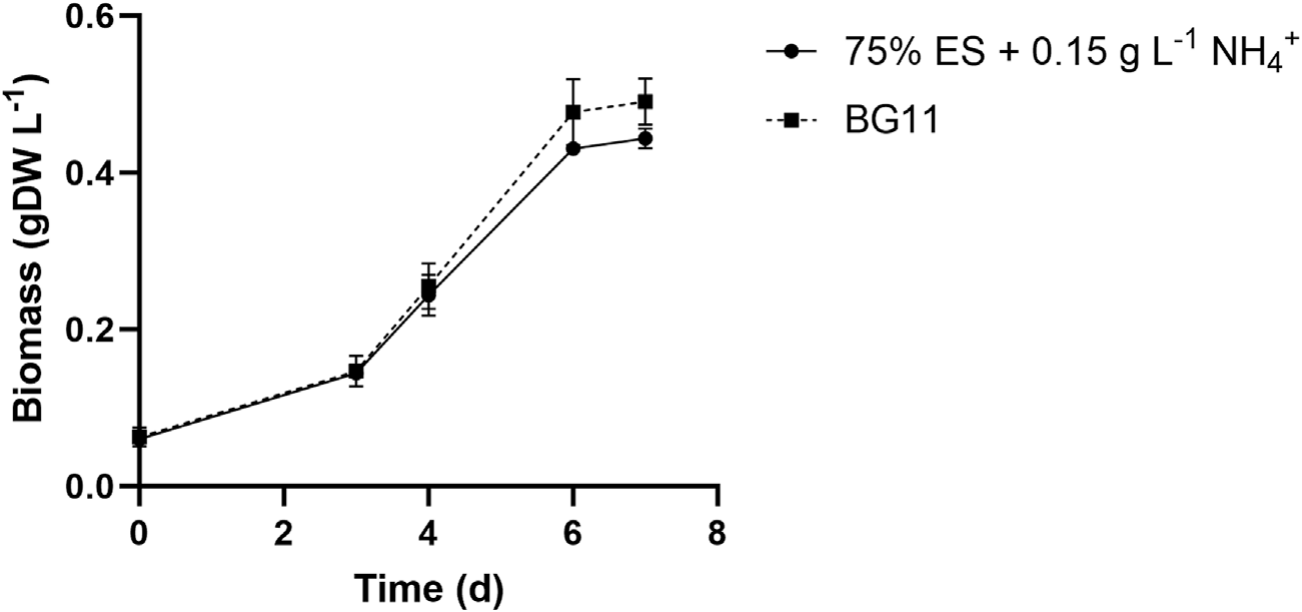
Growth rate of the 2PE_*aroK* mutant on 75% exhausted sludge waste supplemented with 0.15 g L^−1^ NH_4_^**+**^, which was found to be the minimum ammonium concentration required to lead a growth similar to the control condition (BG11). All tests were carried out in triplicate. Error bars represent standard deviation.

The screening took place in 6-well plates, as described in Materials and methods. To qualitatively evaluate the impact of wastewaters for the *S. elongatus* PCC 7942 mutant strain (2PE_*aroK*), a reference condition was adopted, i.e., the bacteria were grown on the BG11 standard medium. Thus, the growth on dairy effluents was assessed as the ratio against the control condition growth, represented by the heatmap in Figure 1, where the orange color represents no growth (equal to Ø) and dark green indicates a growth as the control condition (equal to 1). Furthermore, the percentage indicates the wastewater amount diluted with water (v/v). Thereby, to identify the limiting factor of each wastewater sample, we added singularly or in combination nitrogen (1.5 g L^−1^ NaNO_3_), phosphates (0.04 g L^−1^ K_2_HPO_4_), or trace metal sources at the same concentration as specified for the BG11 medium. Additionally, to assess the effect of nitrogen, phosphates, and trace metals, some negative controls were designed: i) BG11 with no N and P, ii) 75% diluted BG11, and iii) 50% diluted BG11. As theoretically inferred by the chemical nature of CW and Sc, discussed earlier, those wastewaters were detrimental for the cyanobacteria and did not support the growth even when 50% diluted (data not shown). As depicted in Figure 1, the most promising conditions tested were 50%–75% WW and 75% ES. Additionally, for ES, the most limiting nutrient was nitrogen, but supplementing phosphates in combination further improved the cyanobacterial growth. In WW, the deficiency of both metals and nitrogen had the most pronounced effect. To clarify, more diluted conditions were excluded in order to avoid the use of further freshwater and to better align with the concept of wastewaters recycling. The aim of this study was to assess the viability of cultivating our metabolically engineered strain for a sustainable production of 2-PE on wastewaters. Due to the relevance of nitrogen that we found here, we checked for the minimum ammonium concentration that would have guaranteed bacterial growth in ES as in the control condition (BG11). We found that 0.15 g L^−1^ NH_4_^+^ (Figure 2) was the best condition among those studied (0.03–0.15 g L^−1^ NH_4_^+^) for biomass accumulation. Subsequently, to maximize the utilization of wastewaters by the mutant strain, we combined ES and WW in various ratios, carefully considering their chemical features, and conducted a comprehensive examination of pH balance in the following section.

Additionally, the complexity of a growth medium increases as the interaction among all the chemical parts could influence the exploitation by the bacteria. This case in wastewaters should be taken into account.

### 3.3 Coupling dairy wastewaters for nutritional balancing and water recycling

#### 3.3.1 pH tolerance and pH control effect on the 2PE_*aroK* recombinant strain

**FIGURE 3:**
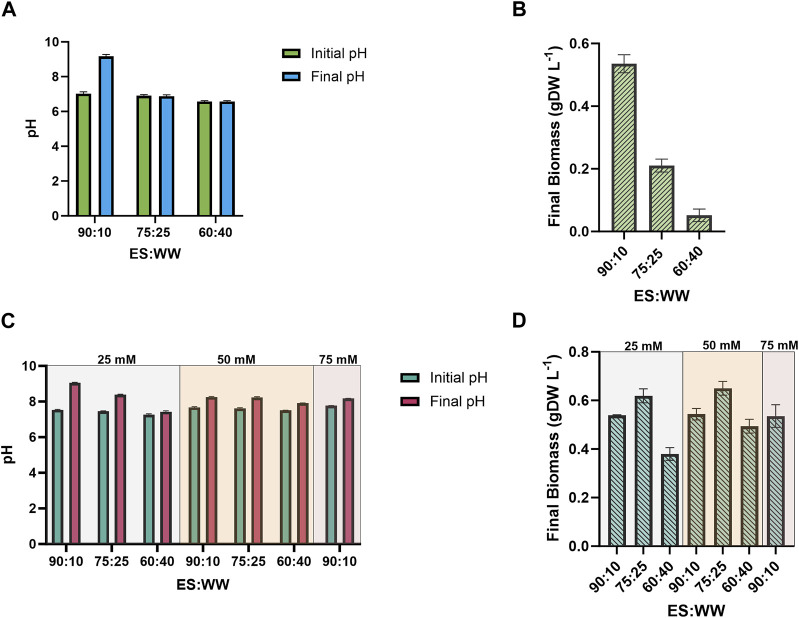
Study of the effect of no pH control and MOPS pH control on the growth performance of the 2PE_*aroK* mutant strain, on different ratios between ES and WW: i) 90:10; ii) 75:25; iii) 60:40. **(A)** pH changes during the cultivation with no pH control. **(B)** Biomass accumulation, expressed as gDW L^−1^, with no pH control. **(C)** pH changes during the cultivation with 25, 50, 75 mM MOPS. **(D)** Biomass accumulation, expressed as gDW L^−1^, with 25, 50, 75 mM MOPS. All tests were carried out in triplicate. Error bars represent standard deviation.

*S. elongatus* PCC 7942 shows optimum growth at pH values ranging from 7.0 to 9.0 (Billini et al., 2008). In the effort to simultaneously balance the pH (at ca. 7.5) and the relative nutritional contribution, we mixed ES and WW in different ratios, and 0.15 g L^−1^ NH_4_^+^ was supplemented in this pool of experiments. The data related to the pH screening are summarized in Supplementary Table S2. Figure 3 shows the outcomes of the wastewater mixing without pH control. The best result was reached at 90 ES: 10 WW, where the initial pH was 7.5, coherently with the optimum for *S. elongatus*. Unfortunately, while the bacteria grew, the pH increased rapidly to a final value of 9.2, which likely inhibited the growth. Conversely, because of the low pH, the mutant strain was not able to grow at the 60:40 ratio (Figure 3B). To better understand the impact of pH on these cultivations, we selected a molecule capable of buffering at the desired pH, i.e., MOPS (range 6.5–7.9). We also evaluated the use of our reference buffer present in our BG11 medium recipe, i.e., TES. However, we found out that TES supplementation led to the formation of particulate, and even if the cyanobacteria could grow (Supplementary Table S2), we decided not to further investigate this option. The MOPS buffer was tested at three different concentrations: 25, 50, and 75 mM (Figures 3C, D). Notably, 25 mM MOPS was able to keep the pH stable in almost all the mixtures tested, and only 90:10 condition required to be buffered at the highest MOPS concentration of 75 mM, with no improvement for cyanobacterial growth (Figure 3D). Both 25- and 50-mM MOPS concentrations efficiently buffered the mixtures between ES and WW, but no quantitative effect on the bacterial growth was recorded, and for this reason, only 25 mM MOPS was selected for further investigations. Regarding the ratio between ES:WW, the 60:40 ratio proved unsuitable for this specific application. On the other hand, 75:25 and 90:10 ratios emerged as the most promising ones and worthy of a deeper study, as discussed in the following section.

### 3.4 Characterization of the 2PE_*aroK* mutant in two mixtures of ES and WW

#### 3.4.1 Growth of the 2PE_*aroK* mutant strain in wastewaters

Although the mixtures designed here contain a small amount of L-Phe (approximately 70 and 25 mg L^−1^ were quantified in the ratios 75:25 and 90:10, respectively), it was not sufficient to support 2-PE production. Usai et al. (2022) extensively described the effect of metabolite doping provided as L-Phe supplementation as necessary for improving 2-PE production by the mutant strain. Thereby, extra L-Phe (0.3 g L^−1^) was added to the wastewaters at the beginning of the test or at the moment of the gene expression induction (triggered by 1 mM IPTG at the 6th day). Four different conditions were thought for both 75:25 and 90:10 ratios. All the conditions contain 25 mM MOPS as the pH buffering system in order to exclude the pH as the object of this investigation.

i. **Control**: The 2PE_*aroK* mutant strain grown with no extra supplementations on ES:WW
ii. **Condition I**: 0.3 g L^−1^ L-Phe was supplemented at 6-day growth
iii. **Condition II**: 0.15 g L^−1^ NH_4_^+^ and 0.3 g L^−1^ L-Phe were supplemented at 6-day growth
iv. **Condition III**: 0.15 g L^−1^ NH_4_^+^ + 0.3 g L^−1^ L-Phe (nitrogen and L-Phe were added as part of the medium at the beginning of the test)

**FIGURE 4:**
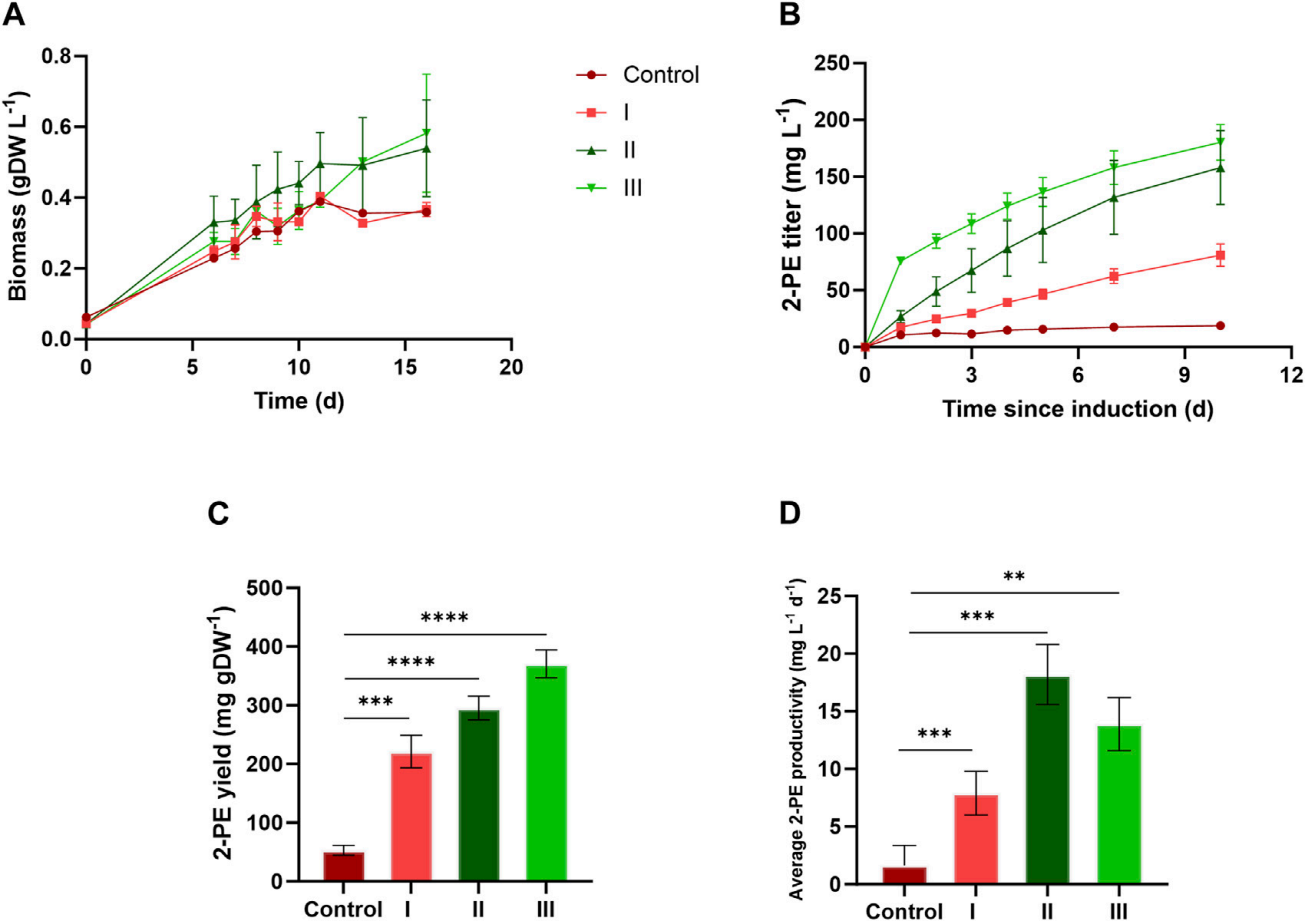
Comparison between 2PE_*aroK* grown on three different conditions on 75 ES:25 WW. **(A)** Growth curves, expressed as biomass (gDW L^−1^). **(B)** 2-PE production kinetics obtained in 10 days since the gene expression induction (mg L^−1^). **(C)** 2-PE yield expressed as mg 2-PE normalized by the biomass produced. **(D)** 2-PE daily productivity (mg L^-1^ d^−1^). All tests were carried out in biological triplicate. Error bars represent standard deviation. The statistical tests were performed as the unpaired *t*-test (α ≤ 0.05) versus the control condition. *, *p*-value ≤ 0.05; **, *p*-value ≤ 0.002; ***, *p*-value ≤ 0.0002; and ****, *p*-value ≤ 0.0001.

**FIGURE 5:**
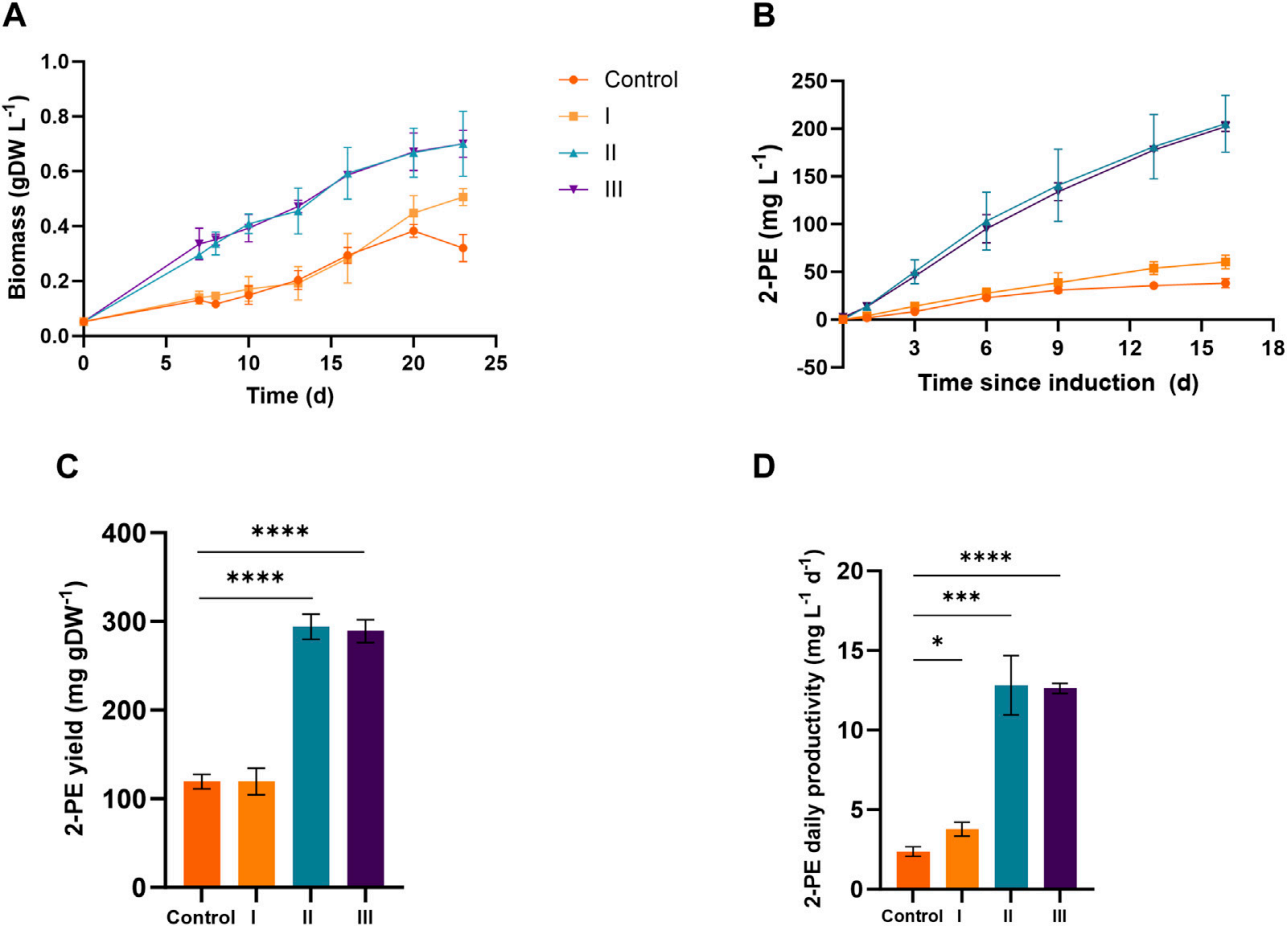
Comparison between 2PE_*aroK* grown on three different conditions on 90 ES:10 WW. **(A)** Growth curves, expressed as biomass (gDW L^−1^). **(B)** 2-PE production kinetics obtained in 16 days since the gene expression induction (mg L^−1^). **(C)** 2-PE yield expressed as mg 2-PE normalized by the biomass produced. **(D)** 2-PE daily productivity (mg L^−1^ d^−1^). All tests were carried out in biological triplicate. Error bars represent standard deviation. The statistical tests were performed as the unpaired *t*-test (α ≤ 0.05) versus the control condition. *, *p*-value ≤ 0.05; **, *p*-value ≤ 0.002; ***, *p*-value ≤ 0.0002; and ****, *p*-value ≤ 0.0001.

Figures 4 and 5 depict the results relative to 75:25 and 90:10 ratios, respectively, regarding the bacterial growth and 2-PE production. The data were collected until the 2-PE synthesis was detected, resulting in a production time of 10 days for 75:25 ratio and 16 days for 90:10 ratio after the induction of the gene expression. All the main findings are summed up in Table 2, where the results of the statistical analysis are reported and performed as multiple *t*-test (unpaired, α ≤ 0.05) versus the control condition. Almost all the results were statistically significant against the control condition (with only some exceptions for the pigment quantification). Significantly, in 75:25 ratio, the specific growth rate (µ, d^−1^) for all the supplemented conditions and control averaged 2-fold higher than those detected in 90:10 ratio (Table 2). Unsurprisingly, nitrogen supplementation positively affected the biomass formation in both the ratios (Figure 4A; Figure 5A; conditions II and III), leading to a biomass accumulation of averaged 0.6 and 0.7 gDW L^−1^ in 75:25 and 90:10 ratios, respectively. On the other hand, the L-Phe addition at the beginning of the test (Figure 4A; Figure 5A; condition III) seemed not to improve the mutant growth.

#### 3.4.2 2-PE production and L-Phe consumption in wastewaters

The 2-PE production ability of the mutant strain in wastewaters was improved compared to the control conditions (no extra supplementations on ES:WW), yielding to 19 and 38 mg L^−1^ at 75:25 and 90:10 ratios, respectively (Figure 4B; Figure 5B). However, at both the ratios, the highest titers were obtained in conditions II and III, accounting for 180 and 205 mg L^−1^ for 75:25 and 90:10 ratios, respectively. In both 90:10 and 75:25 ratios, conditions II and III were not statistically comparable, so the addition of L-Phe at the beginning of the experiment or later on did not improve the bioprocess. On the other hand, the volumetric productivity significantly changed between the two mixtures. A productivity of 12.82 mg L^−1^ d^−1^ was detected in 90:10 ratio (Figure 5D) against 18.03 mg L^−1^ d^−1^ in 75:25 ratio (Figure 4D). This means that the production time in the 90:10 mixture necessary to reach the highest 2-PE concentration is longer (16 days) than that in the 75:25 mixture (10 days).

**FIGURE 6:**
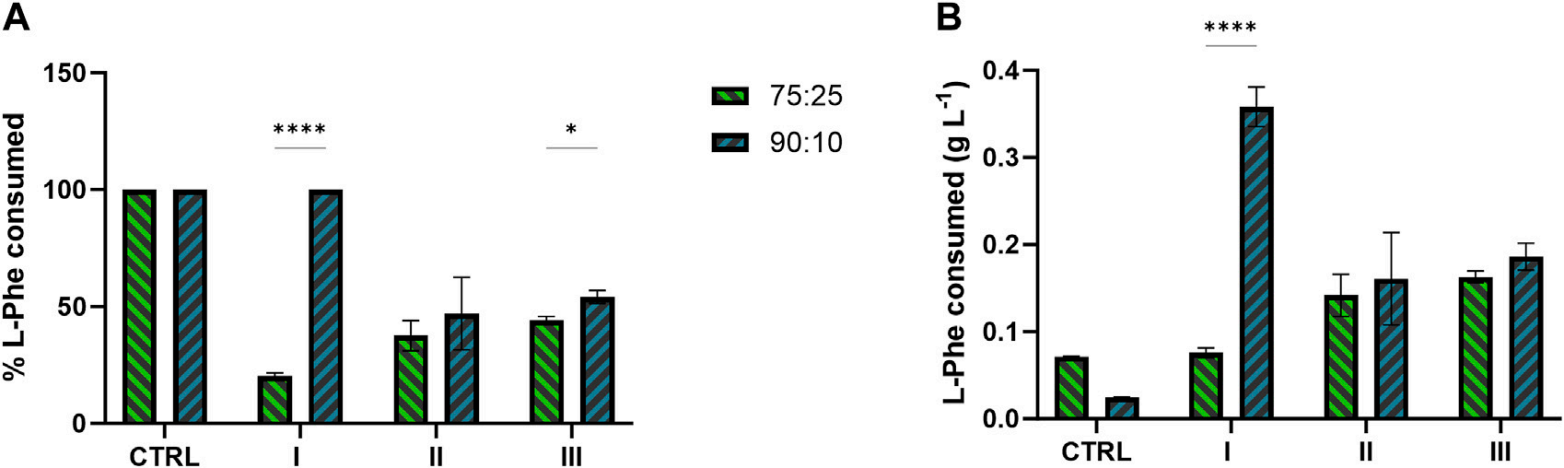
L-Phe consumption by the 2PE_*aroK* mutant strain grown on 75:25 and 90:10 ratios between ES and WW. **(A)** Percentage of L-Phe consumed over the L-Phe available. **(B)** Total amount of L-Phe consumed (g L^−1^) in all the conditions tested and control of the two wastewater mixtures. All tests were carried out in biological triplicate. Error bars represent standard deviation. Asterisks refer to statistical significance (unpaired *t*-test, α ≤ 0.05).

Concerning the metabolite doping, the L-Phe consumption profiled differently between 75:25 and 90:10 ratios (Figures 6A, B). In both the ratios, in conditions II and III, where the L-Phe was supplemented at the 6th day or at the beginning of growth, respectively, a range of 34%–54% (Figure 6A) of the available L-Phe (0.3 g L^−1^ supplemented in addition to the amount already inside) was consumed. Conversely, in condition I (i.e., L-Phe supplementation at 6th day, with no extra N) of the 90:10 mixture, the L-Phe was uptaken by 100% (Figure 6A), with no effect on both bacterial growth and 2-PE production (Figures 5A, B). Additionally, the control conditions for both ratios consumed all the available L-Phe (Figure 6B).

#### 3.4.3 Nutrient removal from wastewaters

**FIGURE 7:**
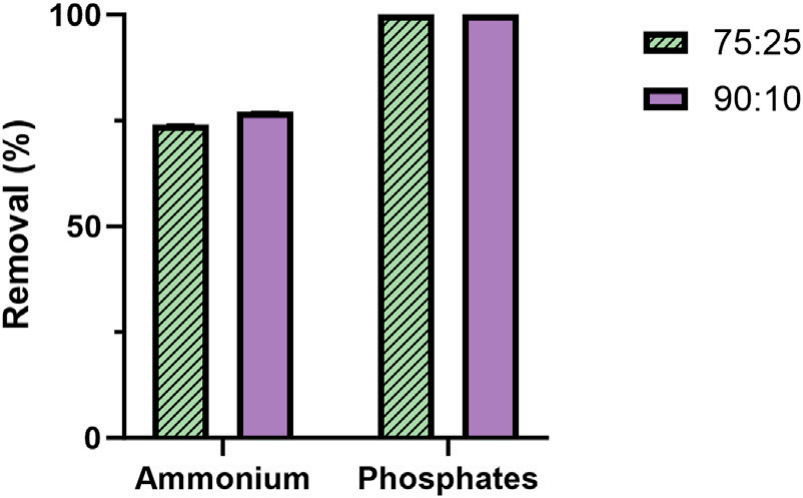
Percentage of N and P removal from the mixture 75 ES:25 WW (patterned) and 90 ES:10 WW (violet). The quantification was carried out in biological triplicate. The error bars represent the standard deviation.

A chemical analysis of the effluent following microbial treatment (Figures 7) was conducted under the two best conditions for bacterial growth and 2-PE production (i.e., ammonium and L-Phe supplementation). N consumption counted for 74% and 77% in 75:25 and 90:10 conditions, respectively. In both the mixtures, P was completely removed (100%).

#### 3.4.4 Photosynthetic pigment accumulation by the 2PE_*aroK* mutant strain in wastewaters

**FIGURE 8:**
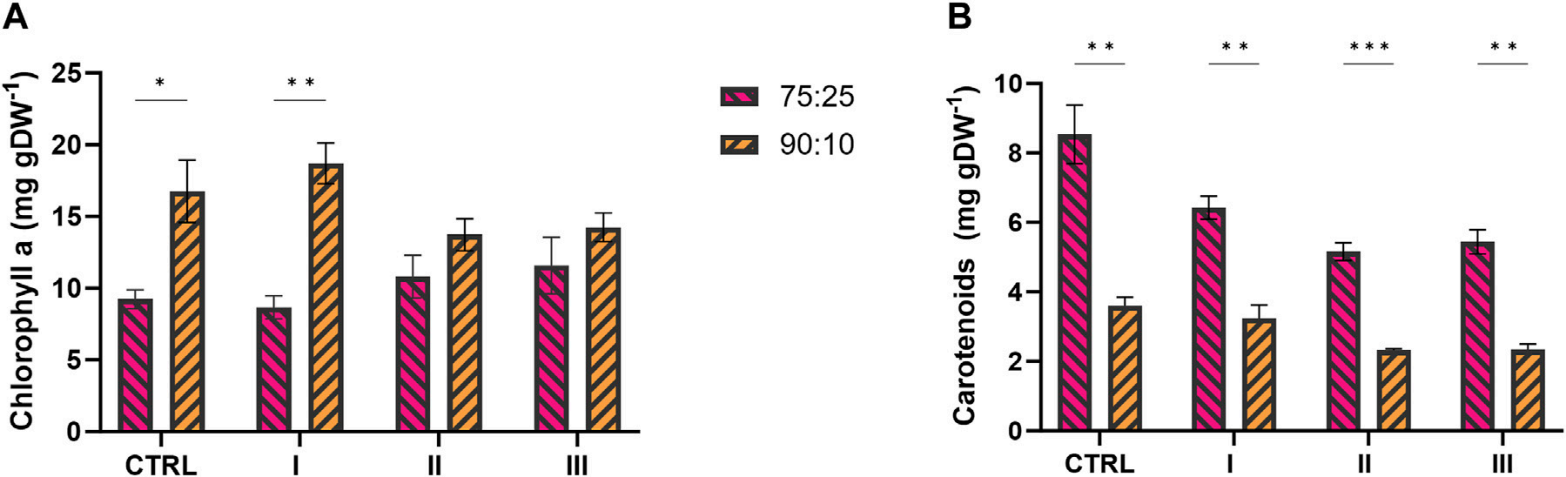
Photosynthetic pigments profile of 2PE_*aroK* mutant strain grown on 75:25 and 90:10 mixtures between ES and WW in three test conditions and control. **(A)** Chlorophyll a yield, expressed as mg gDW^−1^. **(B)** Carotenoids yield, expressed as mg gDW^−1^. All tests were carried out in biological triplicate. Error bars represent standard deviation. The statistical tests were performed as unpaired *t*-test (α ≤ 0.05) *versus* the control condition. *, *p*-value ≤ 0.05; **, *p*-value ≤ 0.002; ***, *p*-value ≤ 0.0002; ****, and *p*-value ≤ 0.0001.

The main photosynthetic pigments, i.e., chlorophyll a (chla) and carotenoids (car), were investigated because they can be used as physiological parameters to assess the quality of the cyanobacterial culture. Figure 8 shows the pigment profile, expressed as mg of pigment per g of dry biomass (mg gDW^−1^), in both 75:25 and 90:10 ratios (see Table 2 for more details). The chla content, measured at the end of the cultivation, was comparable in both 75:25 and 90:10 mixtures (Figure 8A) in conditions II and III, resulting in a range of 11–14 mg gDW^−1^. Between the two ratios, the main difference in chlorophyll content was detected for the control and condition I of 90:10 ratio, where the chla content was almost doubled with respect to that of 75:25 ratio, reaching almost 20 mg gDW^-1^ (Figure 8A). Otherwise, the content of carotenoids was found to be higher in all the conditions exposed to 75:25 ratio instead of 90:10 ratio (Figure 8B). The control condition in the 75:25 ratio showed the highest carotenoid quantification, with 8.5 mg gDW^−1^.

## 4 Discussion

The reuse of wastewaters as nutritional sources for cultivating microorganisms and producing chemicals promotes the concept of a circular economy and ultimately enhances the economic and ecological sustainability of the whole biotechnology. First, to design the sustainable bioprocess, the selection of proper wastewater for specific microorganisms, or vice versa, is the preliminary step, as well as a very challenging task to accomplish. To optimize resource allocation and minimize inefficiencies, it is mandatory to consider the physiological requirements and the final purpose of the relative bioprocess. In this instance, the mutant strain of *S. elongatus* PCC 7942, named 2PE_*aroK*, has been used to evaluate a sustainable 2-PE bioproduction design on dairy wastewaters. The 2PE_*aroK* mutant strain overexpresses five genes, and their rules are summarized in Supplementary Table S1. As already reported (Usai et al., 2022), the 2PE_*aroK* mutant strain needs L-phenylalanine (L-Phe) as metabolite doping to enhance the 2-PE production.

*S. elongatus* is an obligate photoautotroph, and the uptake of organic carbon has been obtained solely by genetic engineering (McEwen et al., 2013; Sarnaik et al., 2017; Sarnaik et al., 2018). However, its excellent ability to rapidly absorb nutrients, such as P and N, simple nutritional requirements, and metabolic plasticity make it a promising biofactory for producing high-value compounds coupled with the treatment of nutrient-rich effluents. The dairy sector generates different types of effluents, which are appealing for biotechnological applications due to the high content of dissolved organic matter and nutrients, such as nitrogen, phosphates, fats, salts, proteins, free amino acids, metals, and minerals, and their inherent composition, including the relative pH, varies based on the unique production line and specific plant-cleaning procedures (Sarkar et al., 2006; Vourch et al., 2008; Carvalho et al., 2013; Licata et al., 2021). Importantly, the need of a substantial volume of freshwater represents a challenge of the milk and cheese industries, resulting in the generation of a considerable excess of wastewater. It has been estimated that the amount of wastewater is one to three times higher than the volume of processed milk (Usmani et al., 2022), and solely in Italy, the effluent volume has been evaluated as 18.9 × 10^6^ m^3^ per year (Stasinakis et al., 2022). Consequently, recovering as much water as possible from the dairy streams should be a main practice toward the water quality and resource management in biotechnology. Different kinds of liquid wastes are produced by dairy industries as primary wastes, such as CW, Sc, and pipelines and tank WWs, or secondary waste such as ES. Early in this study, all the dairy waste streams were considered for this biotechnological application, and at the same time, they were discussed for their employability in microalgae/cyanobacteria-based applications, highlighting the potentialities and challenges. First, they were chemically characterized, and the analysis is reported in Table 1. In the literature, for dairy effluents, the ratio of the key macroelements is reported to be C/N/P ≈ 200/3.5/1, which is generally considered nitrogen deficient to drive any biological processes (Prazeres et al., 2012; Lu et al., 2016). Concerning the nitrogen source, the most used in synthetic cultural media is nitrate (NO_3_^−^). Even if the nitrate uptake is ATP demanding, nitrate is harmless at a high concentration (Flores and Herrero, 1994) and is commonly preferred as nitrogen supply for microbial cultivations. However, in dairy effluents, the most diffused nitrogen form is ammonium (NH_4_^+^), as in several wastewaters (Li et al., 2017). Fortunately, both cyanobacteria and eukaryotic microalgae are able to also assimilate free ammonium as a nitrogen source, but often in wastewaters, the concentration is too high to be tolerated by these microbes. As reported, ammonium would inhibit the photosynthesis of some cyanobacteria species even at ca. 30 mg L^-1^ NH_4_^+^ (Belkin and Boussiba, 1991; Dai et al., 2008). Sometimes, strong dilution (Park and Craggs, 2011; Xia and Murphy, 2016) or nitrification (Sekine et al., 2020; Casagli et al., 2021; Das et al., 2022; Liu et al., 2022) of ammonium-rich wastewaters are crucial prior to feed photosynthetic microorganisms. Nevertheless, here, we quantified a putative deficiency in ready-to-use nitrogen sources, such as NO_3_^−^ and NH_4_^+^. The nitrate ion in the present wastewaters was under the detection limit (<1.5 mg L^-1^ NO_3_^−^). To be precise, the nitrate ion concentration in the BG11 medium is 1.09 g L^−1^. On the other hand, phosphates were sufficient for supporting cyanobacteria growth, even at the lowest concentrations measured (Table 1). High phosphate loads definitely promote the cyanobacterial biomass accumulation (Burberg et al., 2020), as indicated in the context of eutrophication. This phenomenon is often attributed to the high content of phosphates in cleaning agents and fertilizers, and even the influence of climate change can promote phosphates accumulation in waterbodies (Forber et al., 2018; Wilson et al., 2019). Additionally, aquatic habitats are characterized by temporal fluctuations in phosphate availability; so, to cope with this, cyanobacteria evolved several smart strategies to thrive even at low phosphate concentrations (Riegman et al., 2000; Adams et al., 2008; Van Mooy et al., 2009).

Often, the salinity is the main issue affecting the employability of dairy wastes in the non-halotolerant microalgae and cyanobacteria bioprocess, sometimes requiring strong dilutions. On the other hand, the osmotic stress caused by high salt concentration is used as a strategy to boost the high-value carotenoid synthesis in several microalgae, e.g., *Dunaliella salina* and *Haematococcus pluvialis* (Sedjati et al., 2019; Li et al., 2022). Nevertheless, salinity does not appear to be a problem for *S. elongatus* PCC 7942, which has been reported to have a high salinity tolerance of up to 0.4 M (23 g L^−1^) (Dong et al., 2023), which is quite a high value for a freshwater microorganism.

Microalgae and cyanobacteria are proficient in reducing heavy metal content in wastewaters, e.g., chromium, copper, lead, arsenic, mercury, nickel, and cadmium, by bioaccumulation and biosorption (Chakravorty et al., 2023). Instead, many heavy metals, such as zinc, iron, cobalt, and manganese, are crucial for microalgae/cyanobacteria metabolism (Balzano et al., 2020). Metal homeostasis is especially important in cyanobacteria because the photosynthetic machinery imposes a high demand for metals, acting as cofactors of several proteins (Huertas et al., 2014). Even at higher concentrations of some heavy metals, microalgae show elevated biomass, thereby proving to be a suitable candidate for bioremediation (Upadhyay et al., 2016). Here, Cu and Fe were discussed because of their role either in photosynthesis or respiratory electron chains (Shcolnick and Keren, 2006; López-Maury et al., 2012). Only Cu in ES was under the detection limit (Table 1), while in the other wastewaters samples, the concentration of both metals could hypothetically sustain cyanobacteria as well as microalgae cultivation. Notably, pH and COD can strongly influence this type of cultivations (Giraldez-Ruiz et al., 1997). In fact, cyanobacteria show growth at pH values ranging from 7.5 to 11, and they are practically absent in habitats with pH < 5 (Brock, 1973). Moreover, the pH also strongly affects the CO_2_ concentrating mechanism (CCM) (Mangan et al., 2016). Otherwise, some eukaryotic microalgae have been successfully cultivated in very acidic wastewaters (Abinandan et al., 2021; Yu et al., 2022). Given the importance of pH, a detailed investigation of pH effect on our strain is accomplished and will be discussed later on. Following the chemical characterization of the dairy wastewaters, they were qualitatively screened for cyanobacterial growth. This test was conducted on the 2PE_*aroK* mutant strain, which is our chassis for addressing this bioprocess aimed at producing 2-PE biologically. As expected, the qualitative screening highlighted that both CW and Sc were unsuitable for cyanobacteria, even when diluted by 50% (data not shown). Conversely, ES and WW showed that they can support cyanobacteria if opportunely treated and supplemented (Figure 1). The nitrogen source was found to be the main limiting factor in both ES and WW. For this reason, we searched for the minimum ammonium concentration allowing cyanobacteria to grow in 75% ES similarly to the standard medium. We found 0.15 g L^-1^ as the best condition (Figure 2). This is not the first example where nitrogen supplementation was necessary for microalgae applications, especially for secondary wastewater treatments (Huang et al., 2017; Rodrigues-Sousa et al., 2021), such as ES. Additionally, the so-called Redfield ratio (N:P = 16:1) assumes that the balance between nitrogen and phosphates in the medium affects marine photosynthetic microorganisms growth (Redfield, 1934). However, for freshwater microalgae, the Redfield ratio seems to be an exception, and the N:P molar ratios can range between 8:1 and 45:1 with a species-specific preference (Hecky et al., 1993). Here, using ES as the reference, we had N:P ca. 4, which is definitely too low to sustain cyanobacteria. By supplementing extra nitrogen as ammonium, we reached the ratio of 12:26, while in BG11, the N:P molar ratio is approximately 76. Curiously, as reported by Choi and Lee (2015), the biomass production highly depends on the N/P ratio, and they noticed that increase in the ratio up to 10 proportionally promotes the biomass accumulation and then decreases to a constant value, even further raising the ratio. So, theoretically, considering the intrinsic stoichiometry of specific microalgae, the wastewater bioremediation approach could be tailor-made and, thus, optimized to address specific sustainability goals.

To simultaneously balance the pH (ca. 7.5) and the nutritional contribution, as discussed earlier, we mixed ES and WW in different ratios supplemented with 0.15 g L^−1^ NH_4_^+^. We also assessed the evolution of the pH level during the cyanobacterial growth with no pH control system (Figure 3A). The best condition found was the 90:10 ratio, where the highest biomass production was allowed (Figure 3B), but the pH rose rapidly from 7.02 to 9.2, probably preventing further cell accumulation. Thus, to better understand how the pH was impacting the cyanobacterial growth, we used a buffering system, i.e., MOPS (6.5–7.9). Three different concentrations were tested on the three mixtures between ES and WW, but the lowest one (i.e., 25 mM MOPS) was suitable for our purposes, while higher concentrations improved the pH control but did not affect the biomass production (Figures 3C, D). Additionally, we noticed that controlling the pH level improved the biomass formation, mainly at 75:25 ratio (Figure 3D). Thereby, we established the minimal requirements for cyanobacteria growth, and a deeper analysis concerning the target molecule (2-PE) production skills in wastewaters was conducted. Particularly, both 75:25 and 90:10 ratios were selected for the following tests, while 60:40 ratio, once again, resulted in failing cyanobacteria culture. Notably, the implementation of buffers in wastewater treatment bioprocesses is not uncommon, especially when the wastewaters are mixed with standard cultural media, which usually contain buffer agents (Song et al., 2020; Lee et al., 2023). Particularly, this is the case of studies at the laboratory scale, where the pH control relies on the manual addition of buffers or acids/bases. In photobioreactors, managing the pH is a consolidated practice, and nowadays, the on/off strategy is largely exploited. Thereby, the pH is constantly monitored, and when the pH rises beyond a set point (depending on the microorganisms or to avoid ammonia volatilization), the injection of CO_2_ is activated and so the pH will be lowered.

Next, four different conditions were designed for both 75:25 and 90:10 ratios. All the cultivation conditions contain 25 mM MOPS as the pH buffering system, and they were differentiated by the supplementation of ammonium and/or L-Phe. In the control condition (no nitrogen or L-Phe supplementation), both the growth and the 2-PE synthesis were drastically reduced. At 75:25 ratio, the bacterial growth was affected mostly by the nitrogen addition (Figure 4A, conditions II and III), while the L-Phe addition at the beginning of the test (Figure 4A, condition III) seemed not to improve the growth. The same trend was detected in the 90:10 ratio for both growth (Figure 5A) and 2-PE production (Figure 5B) kinetics. However, the specific growth rate (µ, d^−1^) was 2-fold higher in 75:25 ratio than in 90:10 ratio in all the conditions and control (Table 2), although the final biomass accumulation was higher (0.7 gDW L^−1^) than that at 75:25 ratio (0.6 gDW L^−1^).

The 2-PE synthesis was directly improved by the supplementation of L-Phe (either at the 6th day of cultivation or at the beginning of the test) and indirectly due to the addition of extra nitrogen, which increased the biomass accumulation (condition II and III). Compared with the control, in both the wastewater mixtures, the 2-PE production was enhanced 5-fold and 9-fold in 90:10 and 75:25 ratios, respectively. While the highest final 2-PE titer was reached in 90:10 ratio (205 mg L^−1^ in condition II, Figure 5B), the daily productivity decreased (12.8 mg L^−1^ d^−1^, Figure 5D), which is a 30% decrement of the highest one in 75:25 ratio (18.03 mg L^−1^ d^−1^, Figure 4D). Therefore, the production kinetics in the 90:10 mixture necessary to reach the highest 2-PE concentration was longer (16 days) than in the 75:25 mixture (10 days), where 180 mg L^−1^ was achieved. Overall, the 2-PE production on wastewaters recorded here was lowered by 37% (in 75:25) and 28% (in 90:10) in comparison with the highest 2-PE titer reported in the literature for the same mutant strain, but under ideal and synthetic laboratory conditions (Usai et al., 2022).

While the mutant strain was subjected to the metabolite doping carried out by adding L-Phe, the metabolite doper was differentially consumed in the tested conditions (Figures 6A, B). In both the ratios, when the L-Phe was supplemented at the 6th day (condition II) or at the beginning of growth (condition III), the consumption ranged from 34% to 54% (Figure 6A) of the available L-Phe. Interestingly, those test conditions in both the ratios led to the highest 2-PE production of 180 and 205 mg L^−1^ for 75:25 and 90:10 ratios, respectively. Considering the theoretical carbon yield between 2-PE (C8) and L-Phe (C9) of 0.89 molC molC^-1^, the actual amount of L-Phe consumed (Figure 6B) on average (between condition II and III) of 7.28 molC L^-1^ and 9.51 molC L^-1^ (75:25 and 90:10, respectively) may contribute to 59% and 64% of the 2-PE produced in the two mixtures. These findings support the hypothesis (Usai et al., 2022) that L-Phe would not be completely used by the mutant for 2-PE synthesis. The amino acid probably partially helps the 2PE_*aroK* strain to overcome some metabolic constraints, which affect this mutant when overexpressing the 2-PE heterologous pathway. Curiously, when L-Phe was supplemented on the 6th day in the 90:10 mixture (condition I), the uptake accounted for 100%, i.e., 0.347 g L^-1^ (Figure 6A), an outlier of this investigation; however, this massive L-Phe uptake had no effect on both growth and 2-PE production (Figures 5A, B). An analysis of the removal of the main macronutrients, i.e., N and P, confirmed the strong capability of cyanobacteria to phycoremediate wastewaters (Figure 7). All available phosphates were consumed, and ammonium was reduced by 74% and 77% in the 75:25 and 90:10 mixtures, respectively. The present results are perfectly consistent with previous studies conducted in both prokaryotic and eukaryotic microalgae (Kothari et al., 2013; Qin et al., 2014; Choi, 2016; Chokshi et al., 2016; Brar et al., 2019; Kusmayadi et al., 2022; Praveen et al., 2023).

Interestingly, we observed that the photosynthetic pigment production exhibited distinct profiles within the different mixture conditions (Figure 8; Table 2). The assessment of photosynthetic pigments is a common physiological parameter to evaluate the quality of cyanobacteria/microalgae cultures. The chla content, measured at the end of the cultivation, was comparable in both 75:25 and 90:10 mixtures (Figure 8A), while the amount of carotenoids profiled differently between the two wastewater ratios (Figure 8B). Within the two ratios, the main difference in chlorophyll content was found in control condition I, while in 90:10 ratio, the chla content was almost doubled with respect to 75:25 (Figure 8A). On the other hand, the carotenoid content was strongly increased in all the conditions tested for 75:25 ratio (Figure 8B) compared with 90:10 ratio (*p*-values ≤0.002). It is important to stress that all the tested conditions here were subjected to the same environmental parameters (light intensity, temperature, and shaking); thereby, only the medium composition was the study object. Excluding the light as a stress factor affecting the chla content in both the wastewater mixtures, the limiting factor should be searched elsewhere. As reported in the literature, under iron deficiency, the amount of chlorophyll decreases and that of carotenoids increases (Ivanov et al., 2007; Jia et al., 2021). Accordingly, from the chemical analysis reported in Table 1, the iron content in the effluents (42–60 μg L^−1^) was approximately 17-fold lower than that in the standard BG11 medium. This hypothesis could explain the different carotenoid amounts between the two ratios, but, it could not provide any clue about the higher content in the control of 75:25 ratio (Figure 8B). The control condition, actually, may suffer from the strongest stress conditions, where no N or L-Phe supplementation was supplied. Here, several combined stressors could have affected the control growth and pigment accumulations. Even if carotenoids are mainly involved in light-induced stress, they have been found related to any type of stress generating reactive oxygen species (ROS) (Masojídek et al., 2013). Consequently, it is possible that in the 75:25 mixture, where the WW proportion is higher than in the 90:10 mixture, the oxidative effect was more severe due to the higher COD/BOD (Table 1), thus resulting in higher accumulation of carotenoids. Furthermore, this putative oxidative effect in 75:25 ratio seemed not to have any effect on the growth and 2-PE synthesis, which, instead, were improved than those at 90:10 ratio (Table 2). Moreover, the supplementation of N and/or L-Phe appeared to be relieving for this putative oxidative condition. In conditions II and III, the carotenoid content was lower than in the control (Figure 8B) in both 75:25 and 90:10 ratios. Being a wastewater treatment based on photosynthesis, these pigment content-related considerations can help define optimal conditions under which the microorganisms should operate.

However, in scaling up a process, several issues have to be faced. Nutrient-rich effluents are difficult to fully sterilize and suffer from frequent biological contaminations. Nevertheless, some smart strategies have been proposed to allow microalgae and cyanobacteria to compete with contaminants in wastewater treatment as well as in massive volume cultivations. For instance, the recreation of the optimal environment for the target strain, such as stoichiometrically balancing the C/N/P accordingly with the microalgal biomass (Chang et al., 2023), cultivating at high value of pH (Touloupakis et al., 2016; Haines et al., 2022), or salinity (Vonshak et al., 1983; Borowitzka and Borowitzka, 1990). Thus, according to the physiological needs and the chemical features of wastewaters, a process could be designed where the target cultures can thrive. In addition, to reduce biological contaminations for large-volume microalgae cultivation, physical filtration, ultraviolet sterilization, and ultrasound treatment have been considered as effective methods for the *in situ* removal of biological pollutants with insignificant effect on protozoans eggs and spores (Holm et al., 2008; Nawar et al., 2017; Zhu et al., 2020).

Significantly, cultivating engineered cyanobacteria strains in wastewater for sophisticated production is not so common, while wild-type strains are preferred, such as *Spirulina* (Lim et al., 2021; Zapata et al., 2021). Furthermore, traditionally, antibiotics are used as selection markers for recombinant strains and adopted for strain maintenance, as in our case. This is considered as inconvenient for environment-oriented biotechnological approaches, especially in wastewater treatment. Researchers have been focusing on the development of alternative selection markers to avoid antibiotics utilization. For instance, nutritional markers involve mutations in essential metabolic pathways, rendering cells dependent on specific nutrients for growth (Dormatey et al., 2021). Additionally, auxotrophic markers are similar to the previous ones, so the mutant cells can then be selected by their inability to grow in the absence of the required nutrients (Ulfstedt et al., 2017). As the optimal choice, marker-free recombinant strains represent the avant-garde, providing the most environmentally suitable solution (Singh et al., 2022).

Overall, only few reports have been published for chemical production that involves engineered cyanobacteria in wastewater (Hollinshead et al., 2014; Cao, 2019). Thus, this investigation proposes a case study of tertiary treatments via engineered cyanobacteria for sustainable added-value compound bioproduction, which is worthy of further development for larger-volume applications.

## 5 Conclusion

Nowadays, the (almost) unique controversy related to microalgae biotechnology regards the costs of their mass cultivation and the huge volume of water needed. To cut these concerns, many strategies have been proposed among growth medium recycling and the exploitation of wastewaters, which need to be properly treated prior to disposal. As a natural consequence, the utilization of wastewaters in bioprocessing has vast potential for environmental and economic sustainability, aligning with the principles of circular economy, in which resources are recycled and waste minimized.

Here, we propose a sustainable approach to produce 2-phenylethanol using a metabolically engineered cyanobacteria strain (2PE_*aroK*) by combining dairy wastewaters to harmonize microbial physiology with the peculiar composition of these effluents. In this study, the resource optimization for both specific microbial cultivations and sustainability targets is spotlighted. While eukaryotic microalgae support better COD-high dairy wastewater treatments oriented to biomass-related products (e.g., lipids and proteins), cyanobacteria can promote more sophisticated biomanufacturing for high-value compounds even in not-so-rich wastewater, maintaining high growth rates via photoautotrophy. Consequently, freshwater conservation is directly supported, merging the future targets of water management and water quality enhancement. Dairy wastewaters, known for their rich organic carbon content, minerals, nitrogen, and phosphates, are generally considered suitable for microbial cultivation. However, the specific composition of these wastewaters, including pH and nutrient ratios, varies depending on the dairy production process and cleaning procedure. Therefore, efficient utilization of such wastewaters for bioprocessing requires careful consideration of their chemistry. Thereby, we collected some clues aimed at building a tailor-made process to really improve the bioprocess *in toto*. Consequently, by customizing the bioprocess to match the specific composition of different wastewater streams with the biological needs of the microbial chassis, we obtained an effective production of 2-PE on wastewaters, counting for 180 and 205 mg L^−1^ as the best conditions. Thus, these findings ultimately offer a promising case of study for future research and the practical application of sustainable industrial practices, thereby fostering a greener and more responsible approach to photosynthetic biotechnology and bioprocessing.

## Data Availability

The original contributions presented in the study are included in the article/Supplementary Material; further inquiries can be directed to the corresponding authors.

## Abbreviations

BOD, biochemical oxygen demand; Chla, chlorophyll a; CW, cheese whey; Car, carotenoids; COD, chemical oxygen demand; ES, exhausted sludge; 2-PE, 2-phenylethanol; aroK, gene encoding for shikimate kinase; Phe, phenylalanine; Sc, scotta; WW, washing water.
