# Supplementary material for: Coupling dairy wastewaters for nutritional balancing and water recycling: sustainable heterologous 2-phenylethanol production by engineered cyanobacteria
Source: Front Bioeng Biotechnol. 2024 Mar 1;12:1359032. doi: 10.3389/fbioe.2024.1359032 (PMC10940361; doi:10.3389/fbioe.2024.1359032)
Supplement: Supplementary file 1 [file Table1.docx]

**Coupling Dairy Wastewaters for Nutritional Balancing and Water Recycling: A Sustainable Heterologous 2-Phenylethanol Production by Engineered Cyanobacteria**

Giulia Usai^1,2^, Alessandro Cordara^1,3†*^, Elena Mazzocchi^1,2^, Angela Re^2^, Debora Fino^2^, Candido Fabrizio Pirri^1,2^, Barbara Menin^1,4*^

^1^ Centre for Sustainable Future Technologies, Fondazione Istituto Italiano di Tecnologia, Via Livorno 60, 10144, Turin, Italy.

^2^ Department of Applied Science and Technology - DISAT, Politecnico di Torino, Corso Duca degli Abruzzi 24, 10129, 9 Turin, Italy.

^3^ Department of Environment, Land and Infrastructure Engineering – DIATI, Politecnico di Torino, Corso Duca degli Abruzzi 24, 10129, 9 Turin, Italy.

^4^ Istituto di Biologia e Biotecnologia Agraria, Consiglio Nazionale delle Ricerche IBBA-CNR, Via Alfonso 16 Corti 12, 20133 Milan, Italy.

*Correspondence:

Barbara Menin

[barbara.menin@ibba.cnr.it](mailto:barbara.menin@ibba.cnr.it)

Alessandro Cordara

[alessandro.cordara@polito.it](mailto:alessandro.cordara@polito.it)

***Supplementary Materials***

| **Gene** | **HR Site** | **Protein** | **EC number** | **Reaction** | **Organism** | **Ref.** |
| --- | --- | --- | --- | --- | --- | --- |
| aroG^fbr^ | NSI | feedback-inhibition resistant DAHP synthase | EC 2.5.1.54 | PEP + E4P +H_2_O ⇌ DAHP + Pi | *Escherichia coli* | (Ni *et al.*, 2018; Usai *et al.*, 2022) |
| pheA^fbr^ | NSI | feedback-inhibitionresistant chorismate mutase/prephenate dehydratase | EC 5.4.99.5/ EC 4.2.1.51 | Chorismate ⇌ Prephenate ⇌ Phenylpyruvate + H_2_O + CO_2_ | *Escherichia coli* | (Ni *et al.*, 2018; Usai *et al.*, 2022) |
| kivD | NSI | Phenylpyruvate decarboxylase | EC 4.1.1.43 | Phenylpyruvate ⇌ phenylacetaldehyde + CO2 | *Lactococcus lactis* | (Ni *et al.*, 2018; Usai *et al.*, 2022) |
| adhA | NSI | Alcohol dehydrogenase A | EC 1.1.1.1 | Phenylacetaldehyde + NAD(P)H ⇌ 2-Phenylethanol + NAD(P)^+^ | *Synechocystis PCC 6803* | (Ni *et al.*, 2018; Usai *et al.*, 2022) |
| aroK | NSII | Shikimate kinase | EC 2.7.1.71 | Shikimate + ATP ⇌ Shikimate-3-P + ADP | *S. elongatus PCC 7942* | (Usai *et al.*, 2022) |

**Supplementary Table 1. Genetic features of 2PE_aroK recombinant strain of S. elongatus PCC 7942**. HR, homologous recombination; DAHP, 3-deoxy-7-phosphoheptulonate; PEP, phosphoenolpyruvate; E4P, erythrose-4-phosphate.

| **Buffer** | **Molarity** | **ES:WW** | **Initial pH** | **Final pH** | | **Final OD_730_** | |
| --- | --- | --- | --- | --- | --- | --- | --- |
|  |  | **Ratio** |  | **Average** | **SD** | **Average** | **SD** |
| **No Buffer** | **/** | 90:10 | 7.02 | 9.17 | 0.11 | 0.536 | 0.029 |
|  | **/** | 75:25 | 6.9 | 6.88 | 0.07 | 0.210 | 0.021 |
|  | **/** | 60:40 | 6.57 | 6.57 | 0.05 | 0.052 | 0.020 |
| **TES** | **30 mM** | 90:10 | 7.69 | 8.8 | 0.03 | 0.595 | 0.029 |
|  | **30 mM** | 75:25 | 7.56 | 8.6 | 0 | 0.616 | 0.065 |
|  | **30 mM** | 60:40 | 7.41 | 7.96 | 0.03 | 0.510 | 0.026 |
| **MOPS** | **25 mM** | 90:10 | 7.53 | 9.05 | 0.03 | 0.538 | 0.003 |
|  | **25 mM** | 75:25 | 7.45 | 8.38 | 0.03 | 0.619 | 0.029 |
|  | **25 mM** | 60:40 | 7.26 | 7.43 | 0.05 | 0.379 | 0.026 |
|  | **50 mM** | 90:10 | 7.67 | 8.25 | 0.04 | 0.543 | 0.023 |
|  | **50 mM** | 75:25 | 7.61 | 8.23 | 0.04 | 0.650 | 0.028 |
|  | **50 mM** | 60:40 | 7.5 | 7.91 | 0.01 | 0.494 | 0.028 |
|  | **75 mM** | 90:10 | 7.77 | 8.17 | 0.01 | 0.536 | 0.047 |

**Supplementary Table 2. Effect of pH on 2-PE_aroK strain grown on three ES:WW mixtures.** SD, standard deviation. All tests were carried out in biological triplicate. Error bars represent standard deviation.
